# Honey Bee Pollination of *Camellia oleifera* and Mitigation of Toxic Crop Nectar

**DOI:** 10.3390/insects16101028

**Published:** 2025-10-05

**Authors:** Feng Liu, Pingli Dai, Weiliang Zhou, Jinghua Hu, Fang Yuan, Xijian Xu, Wujun Jiang, Qun Luo, Huijun Huang, Ge Zhang, Wuguang Ye

**Affiliations:** 1 Jiangxi Province Key Laboratory of Honeybee Biology and Beekeeping, Apiculture Institute of Jiangxi Province, Nanchang 330052, China; liufeng801012@163.com (F.L.); zhouweiliang9018@163.com (W.Z.); hujinghua93@126.com (J.H.); 18079123732@163.com (F.Y.); xxjlhj2006@163.com (X.X.); jiangwj2260@163.com (W.J.); qunzily1989@163.com (Q.L.); 18979108750@163.com (H.H.); 2 State Key Laboratory of Resource Insects, Institute of Apicultural Research, Chinese Academy of Agricultural Sciences, Beijing 100093, China; daipingli@caas.cn; 3 College of Animal Science and Technology, Shandong Agricultural University, Tai’an 271017, China; 4 Key Laboratory of Efficient Utilization of Non-Grain Feed Resources, Shandong Agricultural University, Tai’an 271017, China; 5 Shandong Provincial Key Laboratory of Animal Nutrition and Efficient Feeding, Shandong Agricultural University, Tai’an 271017, China

**Keywords:** *Camellia oleifera*, *Apis cerana*, *Apis mellifera*, crop pollination, plant toxin, enzyme, detoxification

## Abstract

Tea oil tree (*Camellia oleifera*), a woody oil crop native to Southern China, relies on insect pollination for fruit and seed production. However, its nectar is toxic to honey bees (*Apis* spp.) due to their inability to digest the oligosaccharide present in the nectar. This toxicity raises concerns about the trade-off between the benefits of pollination and the risks posed by exposures to toxic nectar. We aimed to investigate whether tea oil tree yield is enhanced by honey bee pollination, while also examining the impact of nectar toxicity and exploring potential mitigation methods. We evaluated the fruit set, seed yield, and oil quality of the crop with or without eastern honey bee (*A. cerana*) pollination during 2019–2022. We also characterized nectar oligosaccharide compositions collected from both flowers and bee hives. We administered α-galactosidase (an enzyme to promote oligosaccharide digestion) onto bee larvae fed with crop nectar. We found that *A. cerana* could significantly enhance fruit set and seed yield. The administration of α-galactosidase could enhance larval survivorship challenged by nectar toxicity. The effectiveness of honey bee pollination can vary between years, with warmer temperatures significantly enhancing honey bee pollination benefits. The results suggest that a decision to use honey bees for pollinating tea oil trees should involve consideration of the impact of local weather conditions, as low temperatures may compromise pollination benefits while increasing risks posed by toxic nectar. The administration of digestive enzymes to honey bees shows potential for mitigating natural toxins in tea oil tree nectar.

## Simple Summary

Tea oil trees (*Camellia oleifera*) bloom in winter when wild pollinators are scarce. Managed honey bees could provide pollination services at this time; however, the nectar of tea oil trees is toxic to honey bees because they cannot digest its oligosaccharides, hindering sustainable pollination. This study aims to clarify the ecotoxicity of tea oil tree nectar on eastern honey bees (*Apis cerana*) and to propose a mitigation solution for its toxic effect. We found that tea oil tree nectar is toxic to honey bee larvae, but this toxicity can be alleviated by administrating α-galactosidase that can break down oligosaccharides in the digestive system. In addition, we observed that the effectiveness of pollination is affected by annual weather variations, suggesting that honey bee pollination provides greater benefits in favorable years with typically warmer temperatures. Our research offers a promising method for detoxifying this toxic nectar for honey bees, thereby enabling the sustainable pollination of tea oil trees by honey bees.

## 1. Introduction

Tea oil tree (*Camellia oleifera*) is a woody oil-producing crop widely cultivated in southern China (over 4 million ha), and its oil has become a high-quality choice for cooking in China and other countries due to its health benefits, including its high unsaturated fatty acid content (e.g., oleic acid) but low saturated fatty acid content [1,2,3]. Tea oil trees bloom in winter (peaking in November and December), when most animal pollinators are inactive; however, honey bees (*Apis* spp.) are among the few pollinators to facilitate cross pollination during this period [4]. For large pollinating populations and ease of management, growers rent or raise managed western honey bees (*A. mellifera*, an introduced species in China) or eastern honey bees (*A. cerana*, a native species in China) to ensure pollination and higher yield. However, honey bee brood mortality (including larvae and pupae) occurs frequently in tea oil tree orchards [5,6].

Plant metabolites such as phenolics and alkaloids can be toxic to honey bees and human consumers [7,8,9]. Tea oil tree nectar contains oligosaccharides (carbohydrate chain consisting of less than 10 simple sugar units) including maltose, melibiose, manninotriose, raffinose, stachyose, etc., which all show toxicity to honey bee broods [10]. Other oligosaccharides, such as melezitose found in honeydew (sugar secreted from certain insects such as aphids), can also cause poisoning symptoms, including swollen abdomens and impaired mobility as well as disturbing bee gut microbial community [11]. Ingesting oligosaccharides can lead to the death of adult bees by disturbing normal cell function [12].

Oligosaccharides may act as prebiotics to enhance hosts’ intestinal microbial diversity; however, honey bees lack enzymes or intestinal microbes to digest oligosaccharides [11,13,14]. Methods to reduce the exposure to toxic nectar or provide detoxifiers might protect pollinating honey bees in tea oil tree orchards. Enzymes such as α-galactosidase added to human or animal diets can improve the digestion of oligosaccharides when oligosaccharides induce gut health issues [15]. Adding digestive enzymes to honey bees’ diets can be a potential solution to protect bees from toxic nectar from tea oil trees, but this solution needs further validation. In addition to nectar toxicity, tea oil tree pollen contains triterpenoid theasaponins, as one type of phytochemical that can cause honey bee brood mortality [16].

Although honey bees are commonly used for tea oil tree pollination nowadays, there remain fundamental unanswered questions regarding how much seed yield and oil quality is improved by honey bee pollination. Finding an answer to this question becomes more urgent when honey bees are exposed to natural plant toxins, i.e., indigestive oligosaccharides. Honey bees can pollinate tea oil tree flowers; however, the amount of pollen grains transferred from the anther to the pistil by honey bees is lower than that transferred by wild bees, suggesting potentially lower pollination efficiency on a per-flower basis [4]. The honey bee losses (either of individuals or whole colonies) due to nectar toxicity from tea oil tree flowers may exceed pollination benefits if seed yield and oil quality increase is limited. As the tea oil tree industry expands [17], reliance on honey bee pollination will result in more concerns regarding their health.

In this study, we aimed to evaluate the benefits of honey bee pollination of tea oil trees and the risks of exposure to toxic crop nectar, as well as testing potential mitigation methods. We first determined whether native eastern honey bees (*A. cerana*) could enhance tea oil tree productivity by measuring fruit set, seed yield and oil content in orchards over four years (2019–2022, including three growing seasons). To confirm the potential exposure to toxic components of nectar, we collected nectar from tea oil tree flowers and honey from bee hives and analyzed their oligosaccharide contents. We further determined nectar toxicity on eastern honey bee larvae, and then conducted a toxicity mitigation assay by feeding α-galactosidase to larvae. We hypothesized that eastern honey bees would significantly increase fruit set, seed yield, and oil quality, and supplemental feeding of α-galactosidase would rescue eastern honey bee larvae from toxic nectar. Our study assessing tea oil tree production with honey bee pollination will help beekeepers or growers to make appropriate pollinating decisions to minimize the risks from exposure to crop toxins while maximizing pollination benefits. The administration of digestive enzymes provides a potential solution to alleviate the negative impact of pollinating tea oil trees, and this strategy can potentially be applied in other crops or honey plants producing toxic bee food (nectar or pollen).

## 2. Materials and Methods

### 2.1. Experimental Sites and Crops

Three tea oil tree (*C. oleifera*) orchards were selected for this study, Fenjie village (Site 1), Nantang village (Site 2), and Longyuan village (Site 3), all in Xicun Town, Jiangxi Province, in southern China. Site 1 was used for pollination assessment experiments conducted in cages, while all three sites (Sites 1–3) were used for pollination assessment experiments conducted in the open field. The study area has a subtropical climate characterized by ample year-round rainfall, with hot summers but mild winter temperatures (generally 5–26 °C in November and 0–18 °C in December, Figure S1) that can enable tea oil tree flowering in winter. Canola and rice are the dominant crops in the study area, and therefore pesticide use can pose a risk to honey bee health. However, these two crops are harvested before October, when the tea oil trees start blooming, minimizing the likelihood of pesticide exposure from the surrounding crops. No other crops requiring pesticide application were grown within a 2 km radius during the study period.

The tea oil tree fruits developed in winter and spring and were harvested in the fall of the subsequent year. All trees selected for this study were of uniform size and maturity (at least eight years old). Fruit thinning (the removal of extra flowers or fruit) was not conducted, as the study area experiences low fruit set, encouraging growers to retain all the flowers to bear more fruits. Different varieties of *C. oleifera* (including Changlin 4, Changlin 40, and Changlin 53) were grown in the same orchard and assessed in this study. Pesticides were not applied on tea oil trees during fall or winter, when eastern honey bees were present in the orchard. There were no other honey bee hives within a 5 km radius of our research fields.

### 2.2. Evaluation of Honey Bee Pollination of C. oleifera in Cages

Given that low fruit set is the major challenge facing the tea oil tree growing industry in the study area, we focused on the fruit set assessment across all experimental years. Fruit set with and without eastern honey bee pollination was measured in cages installed at Site 1 over three growing seasons (2019–2020, 2020–2021, and 2021–2022) to discover the maximum increase in fruit set by honey bee pollination. The seed yield and oil quality (percentage of total oil and essential fatty acid) were measured specifically in the 2020–2021 growing season.

In the first growing season (2019–2020), six pairs of tea oil trees were randomly chosen at Site 1 (a total of 12 trees studied). Each pair of trees was caged in a 5 m × 5 m net (40-mesh sieve with 0.42 mm diameter that can prevent other smaller pollinators) in later October. At the onset of crop flowering in early November, one eastern honey bee colony was introduced into each of three cages, while the three control cages had no eastern honey bee colonies. Eastern honey bee colonies were established with three frames of adult bees (9000 individuals) and a laying queen bee in each bee hive. In the second (2020–2021) and third (2021–2022) growing seasons, six cages were installed, but number of trees caged in each cage doubled (four tea oil trees per cage), resulting in a total of 24 trees in each season. The honey bee colonies were introduced to the cages in the same way as for the first growing season.

At least a quarter of each tree’s flowers were counted and marked with plastic cards in November of each year (2019, 2020, and 2021). Fruits from marked flowers were counted in July of the following year (2020, 2021, and 2022, respectively) to calculate the fruit set rate. All the fruits from studied trees were harvested in September of the following year for fresh fruit yield and seed yield measurement. Fresh fruits dried naturally in the field until shells and hulls detached with seeds, and both fresh fruit and dried seeds were weighed. The percentage of total oil content and essential fatty acid was analyzed in a government-managed lab: the Forest Product Quality Inspection and Testing Center (Nanchang, Jiangxi province) of the National Forestry and Grassland Administration of China, according to national guidelines [18].

### 2.3. Evaluation of Honey Bee Pollination of C. oleifera in the Open Field

During the 2022–2023 growing season, open-field, commercial-scale pollination was conducted at Sites 1–3. Two honey bee species (western honey bees, *A. mellifera*, and eastern honey bees, *A. cerana*) were used; there were on average nine frames of adult western honey bees (*A. mellifera*), and on average four frames of adult eastern honey bees. The main reason for the difference in population size between the two species in this study is that eastern honey bees usually have a smaller population size than western honey bees due to their biology characteristics. Site 1 had 280 western honey bee colonies to pollinate 630 acres of tea oil trees, and Site 2 had 1200 colonies to pollinate 2550 acres, resulting in a similar density of pollinating colonies at the two sites (0.44 versus 0.47 colony per acre). Site 3 had no honey bee colonies and was considered a control orchard. The commercial colonies were rented from local beekeepers in the study area. Honey bees were moved to orchards when tea oil trees started to bloom in early November (Table S1). There were no other honey bee hives within 5 km radius of our research fields. At each of the three orchards (Sites 1–3), 18, 18, and 12 trees were randomly chosen for assessing fruit set.

### 2.4. Characterization of C. oleifera Nectar and Honey Oligosaccharide Compositions

It remains unclear whether tea oil trees are the sole source of plant toxins in tea oil tree orchards. We tested whether nectar collected from flowers and honey from hives had a similar oligosaccharide profile. Nectar was collected from tea oil trees in the open field at Site 1, apart from the trees used for pollination assessment. Thus, trees and honey bee colonies for pollination assessments would not be disturbed by nectar collection activities. During 3–11 November 2019, 0.25 mL nectar was collected from tea oil tree flowers at Site 1 to assess oligosaccharide composition (refer to Table S2 for nectar sampling information). A flower from each tree was randomly chosen, and the nectar of each flower was repeatedly collected from the nectary using a 50 μL glass capillary tube every 2 h between 7 am and 4 pm.

At the same time, to collect nectar from flowers, tea oil tree honey was collected from additional eight free-flying eastern honey bee hives at Site 1. One empty frame was introduced into each hive during the blooming period, and fresh honey was collected one week later. Gas chromatography/mass spectrometry (GC/MS) was used to identify and quantify 16 oligosaccharides: α, α-trehalose, α, β-trehalose, β, β-trehalose, laminaribiose, nigerose, turanose, maltose, kojibiose, isomalt, gentiobiose, melibiose, isomaltose, raffinose, 1-kestose, erlose, and melezitose (refer to Table S3 for more details of the analysis).

### 2.5. C. oleifera Nectar Toxicity Assessment

The nectar toxicity of tea oil trees was evaluated on eastern honey bee larvae in the laboratory of the Institute of Apicultural Research, Chinese Academy of Agricultural Sciences, Beijing, in 2021. Eastern honey bee queens were caged on empty frames in five hives for 24 h. Five frames with one-day old larvae were pulled out from the hives, and 180 larvae were grafted from frames to 48-well cell culture plates. Larvae from different bee hives were evenly assigned to each treatment group. Larvae were fed with basic larval diets consisting of 6% glucose, 6% fructose, 1% yeast, 50% royal jelly and 37% water for the first two days [19]. From the third day, every three groups of 12 larvae were fed the basic larval diet (control) and four treatment diets, i.e., basic larval diets mixed with each of three honey types (*Vitex negundo*, *Robinia pseudoacacia*, or *C. oleifera*) at a 3:1 ratio by volume, or mixed with 45 mg/L of insecticide dimethoate. The five diets were replenished daily until the sixth day. Larvae were then transferred to new cell culture plates for pupation, and no further feed was provided. The growth and digestive conditions of 6-day old larvae, i.e., body size and amount and color of feces, were visually observed and photos were taken for comparison. The mortality of larvae and pupae was recorded daily for a total of 19 days. The larvae were reared at 32 °C and 60% relative humidity.

### 2.6. Toxicity Mitigation Assays with α-Galactosidase

To address the nectar toxicity of tea oil trees due to their high oligosaccharide content, α-galactosidase was added to larval food to reduce adverse symptoms and enhance the survival rate (assay conducted in Jiangxi Apicultural Institute, Nanchang, in 2021). Each of the three groups of 24 3-day-old larvae were fed one of four diets. These four diets included a basic larval diet (control), a diet containing tea oil tree nectar (basic larval diet and tea oil tree nectar at a ratio of 3:1 by volume), a diet containing tea oil tree nectar and 0.05 mg/mL α-galactosidase, and a diet containing tea oil tree nectar and 0.1 mg/mL α-galactosidase. A total of 288 larvae were tested for the above four diets.

### 2.7. Statistical Analysis

All statistical analyses were performed using R software (version 4.2.1., R Core team, 2023). Fruit set, yield (fruit and seed weight), and oil quality (oil content and essential fatty acid content) was compared between tea oil trees pollinated with and without *A. cerana* over three growing seasons (2019–2020, 2020–2021, 2021–2022) using a Mann–Whitney U test (“wilcox.test” function). To compare honey bee survivorship (immature larvae and pupae) from different diet treatments, a log-rank test was conducted through the “survival” package (“survfit” function and “survdiff” function for pairwise comparisons, with Benjamini–Hochberg adjustment). The Kaplan–Meier survival curve was plotted with the “ggsurvplot” function of the “ggplot” package. When assessing the mitigation effects of α-galactosidase, the survival rate of diet treatments with or without α-galactosidase was compared using ANOVA (“aov” function) and Tukey’s multiple comparisons (basic “TukeyHSD” function).

## 3. Results

### 3.1. Fruit Set, Yield and Oil Quality of C. oleifera with Honey Bee Pollination in Cages

**Figure 1:**
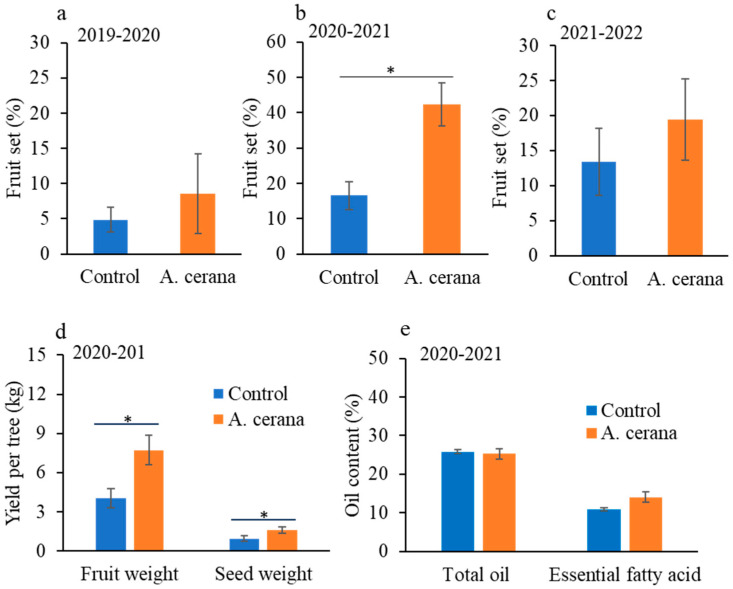
Tea oil tree fruit set, yield and oil quality with pollination by *A. cerana*. Fruit set (mean ± standard error, SE) in 2019–2020 (**a**), 2020–2021 (**b**) and 2021–2022 (**c**). Fresh fruit and dry seed yield (mean ± SE, kg) per tree in 2020–2021 (**d**), and total oil content (mean ± SE, %) and total essential fatty acid content (mean ± SE, %) in 2020–2021 (**e**). Asterisks (*) indicate significant differences between findings with and without eastern honey bee pollination.

The fruit set and yield were improved by *A. cerana* pollination in cages. Fruit set was significantly enhanced by eastern honey bee pollination during 2020–2021 (W = 136, *p* < 0.001; Figure 1b). The increase in fruit set with honey bee pollination was only numerical and not significantly different during 2019–2020 (W = 19, *p* = 0.537) and 2021–2022 (W = 83, *p* = 0.316) (Figure 1a,c).

Both yield parameters (fresh fruit weight and dry seed weight in kilogram) significantly increased with eastern honey bee pollination (fresh fruit weight: W = 119.5, *p* = 0.001; dry seed weight: W = 116, *p* = 0.010) during 2021–2022 (Figure 1d). The percentage of total oil content and essential fatty acids extracted from tea oil tree seeds did not significantly differ between trees with and without eastern honey bee pollination (total oil content: W = 51, *p* = 0.237, Figure 1e; essential fatty acids: W = 28, *p* = 0.132, Figure 1e).

### 3.2. Fruit Set of C. oleifera with Honey Bee Pollination in the Open Field

**Figure 2:**
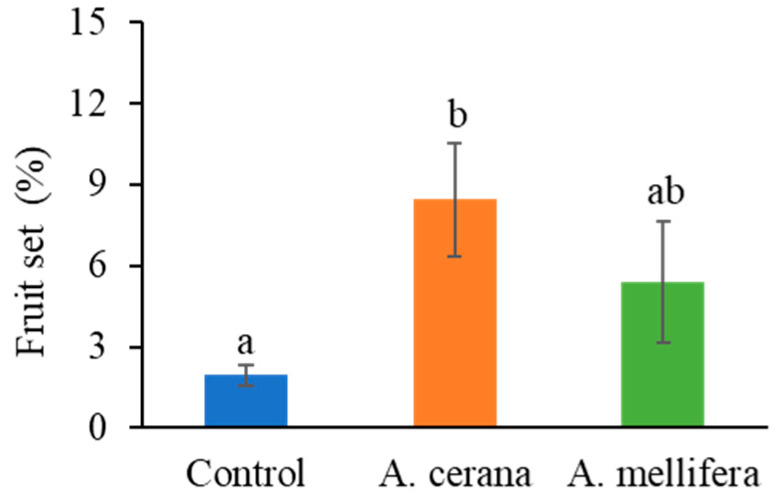
Fruit set of tea oil trees pollinated in the open field with and without honey bee pollination. Different lowercase letters on bars indicate statistical differences.

Fruit set was significantly improved by eastern honey bee pollination in the open field during 2022–2023 (F_2,23_ = 6.624, *p* = 0.004, multiple comparisons with TukeyHSD; Figure 2). Fruit set with pollination by western honey bees (*A. mellifera*) was not significantly different from the control (no honey bee pollination) or eastern honey bee pollination groups (Figure 2).

### 3.3. Profile of C. oleifera Nectar and Honey Oligosaccharides

**Figure 3:**
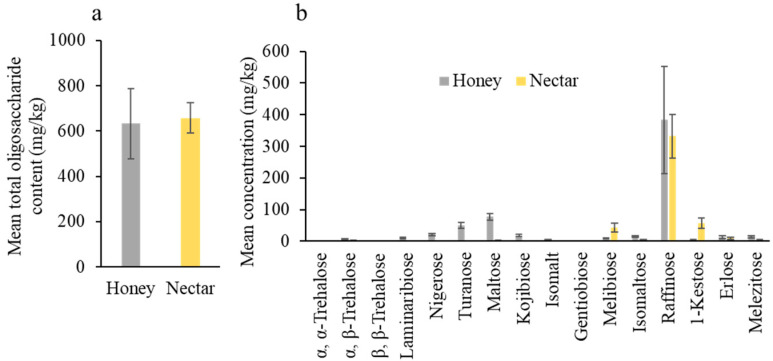
Oligosaccharide compositions of tea oil tree nectar and honey. (**a**) Total oligosaccharide content (mean ± SE, mg/kg) and (**b**) concentration of each oligosaccharide (mean ± SE, mg/kg).

Nectar collected from crop flowers and honey collected from bee hives shared similar oligosaccharide compositions. Total oligosaccharides (<1000 mg/kg or 1%) were not significantly different between nectar collected from tea oil tree flowers and honey collected from pollinating bee colonies (*t* = 1.027, df = 9.612, *p* = 0.330; Figure 3a). The dominant oligosaccharide was raffinose, and there was not a significant difference between crop nectar and honey for each individual oligosaccharide (Figure 3b).

### 3.4. Assessment and Mitigation of C. oleifera Nectar Toxicity

**Figure 4:**
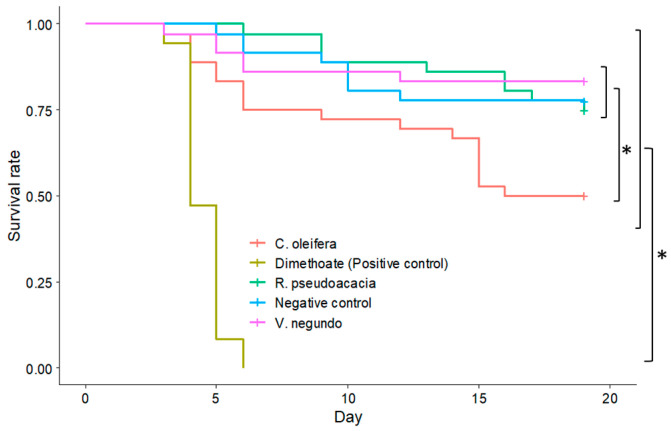
Survival rate (%) of *A. cerana* larvae treated with five diets. The tracking of larval and pupal survival started from 1-day-old larvae and continued for 19 days. Asterisks (*) indicate significant differences between different diet treatments.

*C. oleifera* nectar was found to be toxic to eastern honey bee larvae; however, α-galactosidase proved effective in mitigating its toxicity. Eastern honey bee larvae fed with *C. oleifera* nectar had a significantly lower survival probability than larvae fed with cane sugar solution as a negative control (χ^2^ = 168, df = 4, *p* < 0.001, multiple comparison with Benjamini–Hochberg adjustment; Table S4; Figure 4). The survival probability of larvae fed with *V. negundo* and *R. pseudoacacia* (acacia) honey was not significantly different from that of the control larvae (Table S4; Figure 4). The positive control treatment with insecticide dimethoate killed all the larvae on the fifth day, leading to a significant difference from all other diet treatments (Table S4; Figure 4).

**Figure 5:**
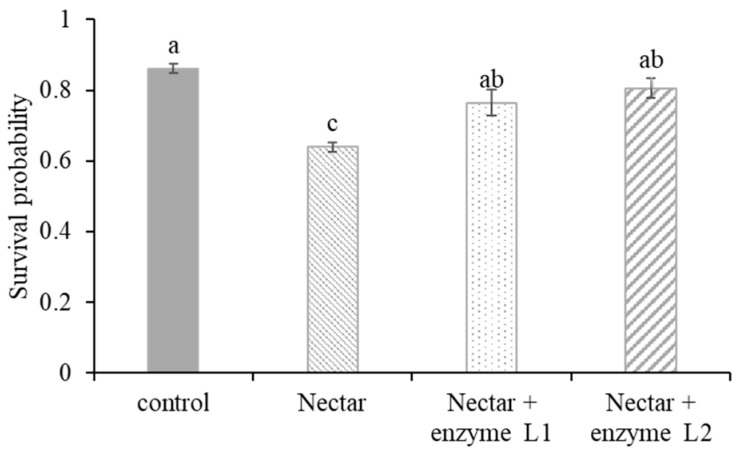
Survival probability (mean ± SE) of 6-day-old larvae (*A. cerana*) treated with *C. oleifera* nectar or a combination of *C. oleifera* nectar and α-galactosidase. Two enzyme α-galactosidase levels (L): L1 at 0.5 mg/mL and L2 at 1 mg/mL. Different letters on error bars indicate statistically significant differences.

**Figure 6:**
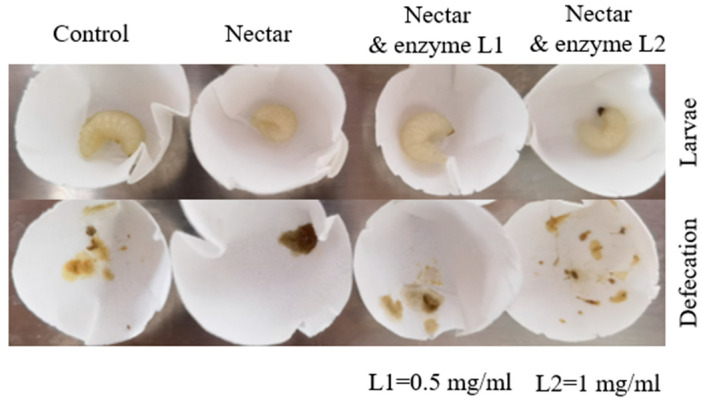
Body size and defecation of larvae (*A. cerana*) treated with *C. oleifera* nectar and/or enzyme. Body size of larvae fed with *C. oleifera* nectar is smaller than larvae fed with control diet and diets containing both nectar and enzyme, α-galactosidase (level 1 at 0.5 mg/mL and level 2 at 1 mg/mL). Larvae fed with *C. oleifera* nectar discharged more and darker feces compared to those taking the control diet and the diet containing enzymes.

In a separate assay to test the effects of α-galactosidase (at either levels of 0.5 mg/mL or 1 mg/mL), the survival rate of larvae fed with α-galactosidase significantly increased compared to those fed with tea oil tree nectar only (F_3,8_ = 14.230, *p* = 0.001, multiple comparison with TukeyHSD; Figure 5). The body size of larvae fed with tea oil tree nectar was smaller than that of larvae fed with a normal diet (control). Larvae fed *C. oleifera* nectar discharged darker, abnormal feces in greater quantities (Figure 6). Both the body size and feces appearance of larvae fed with α-galactosidase at two levels were comparable to those from the control group (Figure 6).

## 4. Discussion

Our study found that eastern honey bees (*A. cerana*) significantly enhanced fruit set in one growing seasons (2020–2021) when trees and bees were caged together, with two other seasons showing a numerical increase in fruit set (no statistically significant difference). Yearly variances in fruit set may be attributed to tree phenology, e.g., the alternate bearing phenomenon, with one year producing a higher yield but the following year producing a lower yield. Weather conditions (temperature, humidity, rainfall, light, etc.) or climate can affect plant–pollinator interactions and thereby plant reproduction, including fruit set [20,21,22,23]. In growing seasons with significantly higher fruit set (2020–2021), we found higher average monthly temperatures in November (the primary blooming period in the study area), suggesting a potential correlation between weather conditions and pollination effectiveness (Figure S1).

During 2020–2021, pollination by eastern honey bees significantly enhanced fruit set under both caged and open field conditions. The increase was more pronounced in cages, with fruit set rising from 1.96% to 8.44% in the open field, compared to an increase from 16.53% to 42.34% within cages. This contrast was caused by the high density of pollinating forager bees in cages. Our results suggest that eastern honey bees are more efficient pollinators than western honey bees. Eastern honey bees have also been found to be more efficient in pollinating other crops such as blueberries grown in a greenhouse [24]. The number of pollen grains deposited onto the stigma determines pollination effectiveness [25], and eastern honey bees can deposit more pollen onto the stigma than western honey bees, such as while pollinating oilseeds, *Brassica napus* [26]. Honey bees (both eastern and western honey bees) are efficient pollen collectors [4,27]; however, eastern honey bees spend less time than western honey bees during each flower visit (e.g., for flower handling or grooming) [26,28]. When spending less time per flower visit, pollen grains are less likely to be compacted tightly on the corbicula and the body surface, resulting in more loose pollen dropping onto the stigma [28]. We did not measure pollen deposition on stigma of tea oil trees, but we observed that eastern honey bees visited more flowers per minute compared to western honey bees, suggesting higher pollination efficiency by eastern honey bees.

Eastern honey bees in a tea oil tree orchard can forage both tea oil tree flowers and other plant flowers; therefore, other plants are also potential causes of larval mortality in these orchards. Analyzing the oligosaccharide compositions of honey collected from bee hives and nectar collected from tea oil tree flowers would confirm that tea oil trees were the source of this plant toxin, unless other plants in the area also produce nectar containing oligosaccharides. The toxicity results in this study aligned with toxic symptoms observed by beekeepers [5] and assays conducted on western honey bee species (*A. mellifera*) [10].

Despite the toxicity of nectar, honey bees are still brought to tea oil tree orchards for pollination to increase yield. The challenges of exposure to negative biotic factors and the demand for honey bee pollination are also evident in other bee-dependent crops, such as apple, blueberry, and almond, where pesticide use, poor forage nutrition, and/or diseases cause significant health issues to bees [29,30,31,32]. With the risk of nectar toxicity, the variance in fruit set between years can be a factor for growers or beekeepers to consider when deciding whether to pollinate tea oil trees or not. For a year with higher fruit set, it would be more favorable; for example, there was a 25.82% increase in fruit set during 2020–2021. However, the insignificant increase in fruit set in the growing seasons of 2019–2020 and 2021–2022 does not encourage bee pollination when the potential yield enhancement with honey bee pollination is likely to be outweighed by bee losses caused by nectar toxicity (Figure 1).

Tea oil tree nectar has been found to be toxic to the larvae of eastern honey bees due to its high oligosaccharide content. The enhanced survival rate when treating honey larvae with α-galactosidase in our lab study suggests that applying α-galactosidase or other potential detoxifying enzymes could serve as a potential solution for addressing nectar toxicity in honey bees. But the dosage and application methods of the enzyme (such as spraying it in the brood area or adding it to supplementary feeding) should be re-evaluated during field use, as colony sociality can affect detoxification efficacy [33]. Our finding of α-galactosidase as an effective detoxifier was also supported by observations of defecation features: abnormal black and chunky feces disappeared when enzyme treatments were given to larvae. Wild bees are more tolerant to oligosaccharides and serve as effective pollinators for tea oil trees [34,35]. However, their natural populations might be insufficient to meet the pollination demand for the large-scale monoculture plantation of tea oil trees. Wild bee pollinators will be particularly valuable for small farms and can complement honey bee pollination in larger agricultural settings.

Exploring appropriate physical beekeeping management practices to mitigate nectar toxicity will be another strategy when enzyme treatment may be effective but costly and labor intensive. Since consuming toxic nectar is the primary cause of bee mortality, it is worthwhile investigating methods to minimize the intake of tea oil tree nectar. Maintaining enough honey storage in bee hives or feeding syrup (artificial food) to the entire colony may dilute toxic nectar or lead to fewer nectar-foraging activities, thus reducing the possibility of acute poisonings caused by nectar. Removing tea oil tree pollen from returning foraging bees with pollen traps might encourage more pollen foraging activities but reduce the inflow of toxic pollen [36,37]. The installment of pollen traps would remove pollen collected from other plants that can cause nutrition shortage, and thus minimum supplementary protein diets should be provided to those pollinating colonies installed with pollen traps. The negative impacts of natural plant toxins within nectar occur in many other bee-dependent crops or wild honey plants such as jujube and blueberry [7,8,38,39]. The manipulation of foraging activities may therefore allow foraging on toxic nectar to be avoided.

In conclusion, we found variations in pollination benefits in tea oil tree orchards that should be considered for honey bee health risk management. The administration of a digestive enzyme, α-galactosidase, is a promising solution to bee poisonings due to the toxic nectar of tea oil trees, and a re-assessment of this solution in realistic field conditions needs be further investigated in future studies. Due to the potential high cost of administration of digestive enzymes, we suggest alternative solutions such as increasing honey and syrup availability to dilute toxic nectar and removing pollen from returning forager bees with pollen traps.

## Data Availability

Data will be shared upon request.

## Supplementary Materials

The following supporting information can be downloaded at: https://www.mdpi.com/article/10.3390/insects16101028/s1, including detailed sample collection, chemical analysis, statistics and climate of the study area. Figure S1: Temperatures during tea oil tree blooming periods across years in the study area; Table S1: Pollination duration by honey bee colonies; Table S2: Nectar collection information used for oligosaccharide composition analysis; Table S3: Standard curve for oligosaccharide measurement; Table S4: Multiple comparison of different diet treatments using Log-Rank test.
