# Supplementary material for: Honey Bee Pollination of Camellia oleifera and Mitigation of Toxic Crop Nectar
Source: Insects. 2025 Oct 5;16(10):1028. doi: 10.3390/insects16101028 (PMC12563362; doi:10.3390/insects16101028)
Supplement: Supplementary file 1 [file insects-16-01028-s001.zip › insects-3866474-supplementary.pdf]

Supplementary Materials

Table S1. Pollination duration by honey bee colonies.

Year	Move-in date	Move-out date [a]
2019	Nov. 11th	Dec. 22nd
2020	Nov. 1st	Dec. 29th
2021	Nov. 3rd	Dec. 12th
2022	Nov. 3rd	Dec. 31st

a. The move-out date was after tea oil tree blooming.

Table S2. Nectar collection information used for oligosaccharide composition analysis.

Date (year-month-day）	Weather type	Temperature (℃)	Humidity（%）	Nectar water content (%)
2020-11-06	Sunny	7.0~13.2	78~96	53.66±2.91
2020-11-07	Light rain	5.0~9.7	76~85	48.20±0.13
2020-11-03	Cloudy	3.0~12.0	63~80	53.34±0.14
2020-11-04	Cloudy to sunny	3.0~14.0	45~65	52.78±0.13
2020-11-05	Sunny	5.0~11.5	67~71	52.31±0.57
2020-11-10	Sunny	9.1~15.0	45~88	51.85±0.71
2020-11-11	Cloudy	7.0~13.2	78~96	55.93±1.59
2020-11-08	Cloudy	5.0~9.7	76~85	54.13±3.17
2020-11-09	Sunny	8.2~14.0	73~81	48.91±0.96

Table S3. Standard curve for oligosaccharide measurement.

Chemical name	Regression equation	Range (μg/ml)	Correlation coefficient (R)	LOD (g/100 g)[a]	LOQ (g/100 g)
Sucrose	y = 0.0334x + 0.0237	0.5-80	0.9988	0.010	0.05
α, α-Trehalose	y = 0.0227x - 0.0035	0.5-80	0.9995	0.005	0.01
α, β-Trehalose	y = 0.0187x + 0.0008	0.5-100	0.9995	0.005	0.01
β, β-Trehalose	y = 0.0246x - 0.0076	0.5-80	0.9996	0.005	0.01
Laminaribiose	y = 0.0413x - 0.0223	0.5-80	0.9984	0.005	0.01
Nigerose	y = 0.0374x - 0.049	0.5-100	0.996	0.005	0.01
Turanose	y = 0.0479x - 0.0467	0.5-100	0.9989	0.005	0.01
Maltose	y = 0.0258x - 0.0219	0.5-80	0.9993	0.010	0.02
Isomalt	y = 0.036x - 0.0225	0.5-80	0.9991	0.010	0.05
Gentiobiose	y = 0.0828x - 0.0458	0.5-80	0.9996	0.010	0.05
Melibiose	y = 0.0251x - 0.0147	0.5-80	0.9993	0.010	0.05
Isomaltose	y = 0.0463x - 0.0178	0.5-80	0.9997	0.010	0.05
Raffinose	y = 0.1413x - 0.1386	0.5-80	0.9966	0.005	0.01
1-Kestose	y = 0.2509x - 0.2002	0.5-50	0.9973	0.010	0.05
Erlose	y = 0.0377x - 0.0377	0.5-50	0.9953	0.010	0.02
Melezitose	y = 0.0258x - 0.0181	0.5-80	0.999	0.005	0.01

a. Limit of detection, LOD; limit of quantification, LOQ.

Table S4. Multiple comparison of different diet treatments using Log-Rank test.

Treatment	*C. oleifera*	Dimethoate (positive control)	*R. pseudoacacia*	Sugar (negative control)
Dimethoate (positive control)	<0.0001			
R. pseudoacacia	0.0246	< 0.0001		
Sugar (negative control)	0.0246	< 0.0001	0.8819	
V. negundo	0.0092	< 0.0001	0.6166	0.689

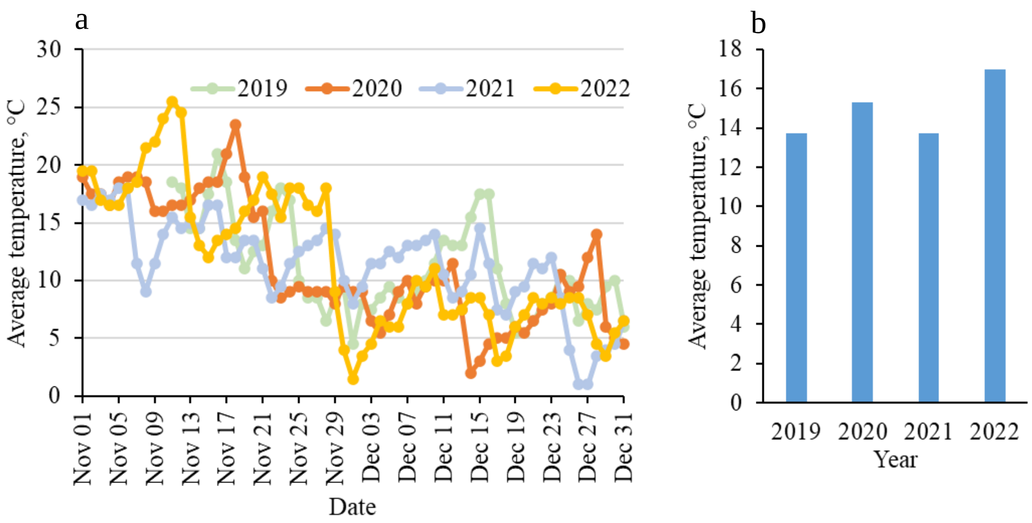

Figure S1. Temperatures during tea oil tree blooming periods across years in the study area. (a) November and December daily average temperatures during four years (2019-2022). (b) November monthly average temperatures.
